# Levodopa-induced motor complications associated with benserazide and carbidopa in Parkinson’s disease: a disproportionality analysis of the FAERS database

**DOI:** 10.3389/fphar.2025.1529932

**Published:** 2025-03-06

**Authors:** Huaide Qiu, Cheng Liu, Zhixiang Wang

**Affiliations:** ^1^ School of Rehabilitation Science, Nanjing Normal University of Special Education, Nanjing, China; ^2^ Department of Rehabilitation Medicine, Yixing No. 2 People’s Hospital (Yixing Prevention and Treatment Hospital for Occupational Diseases), Yixing, Jiangsu, China; ^3^ Rehabilitation Medicine Center, The First Affiliated Hospital of Nanjing Medical University, Nanjing, China

**Keywords:** Parkinson’s disease, motor complications, levodopa, benserazide, carbidopa

## Abstract

**Background:**

Levodopa-induced motor complications are a significant concern in the treatment of Parkinson’s disease (PD). Dopamine decarboxylase inhibitors (DCIs) such as benserazide (BSZ) and carbidopa (CD) are commonly used in conjunction with levodopa to manage PD symptoms. However, their association with motor complications remains unclear.

**Methods:**

We performed a retrospective pharmacovigilance analysis using the FDA Adverse Event Reporting System (FAERS) data from Q1 2004 to Q2 2024. The study included only adverse event reports (AERs) related to oral administration of drugs indicated for PD. We concentrated on motor complications, selecting two system organ classes (SOCs) associated with motor fluctuations and dyskinesia: nervous system disorders and general disorders/administration site conditions. Disproportionality analysis and Bayesian methods were utilized to identify and assess motor complication signals associated with BSZ and CD. A signal was deemed significant if it met the following criteria: reporting odds ratio (ROR) ≥ 3 with a 95% confidence interval (CI) lower bound >1, information component (IC) 95% CI lower bound >0, and empirical Bayes geometric mean (EBGM) 95% CI lower bound >2.

**Results:**

The analysis identified 8,744 AERs related to motor complications, recording 19,482 adverse events (AEs). The study highlighted motor complications such as dyskinesia, the on-off phenomenon, freezing episodes, and wearing-off, linked to the oral use of both BSZ and CD. Dyskinesia showed high RORs for both BSZ (16.5, 95% CI 14.76–18.45) and CD (13.81, 95% CI 13.02–14.65). The on-off phenomenon demonstrated a more pronounced ROR for BSZ at 170.74 (95% CI 145.03–201.01) compared to CD at 67.5 (95% CI 59.46–76.63). Wearing-off was notably higher for CD, with an ROR of 7.66 (95% CI 7.08–8.28), compared to BSZ’s ROR of 3.03 (95% CI 2.37–3.88).

**Conclusion:**

The findings indicate that the choice of DCI affects the risk profile of motor complications in PD. BSZ is associated with increased risks of dyskinesia and the on-off phenomenon, whereas CD is linked to a higher risk of wearing-off. Future research should explore the mechanisms underlying these differences to guide the selection of the most appropriate DCI for individual patients.

## 1 Introduction

Parkinson’s disease (PD) is a progressive neurodegenerative disorder that is pathologically defined by the loss of dopaminergic neurons within the substantia nigra, along with the formation of Lewy bodies (Olanow et al., 2009). The neuronal depletion leads to a significant reduction in the neurotransmitter dopamine (DA), which causes a range of hallmark motor symptoms in individuals with PD, including tremor at rest, bradykinesia, muscular rigidity, and postural instability (Armstrong and Okun, 2020). These symptoms progressively worsen over time, significantly impairing the quality of life and daily functioning of affected individuals. Globally, PD affects over six million people and is a leading cause of disability, imposing a substantial burden on healthcare systems and society at large (Dorsey et al., 2018).

The cornerstone of PD management has long been the administration of levodopa (LD), a precursor to dopamine (Connolly and Lang, 2014). In the bloodstream, levodopa is primarily converted into dopamine and 3-O-methyldopa through the actions of decarboxylase and catechol O-methyltransferase (COMT), respectively. As a result, a mere 1% of orally administered levodopa reaches the dopaminergic neurons (Jankovic, 2002; Contin and Martinelli, 2010). To address this, levodopa is often given in combination with a decarboxylase inhibitor (DCI), such as benserazide (BSZ) and carbidopa (CD), in a single tablet form (Tolosa et al., 1998). This combined administration enhances the availability of levodopa in the brain by ten-fold and extends its peripheral half-life to roughly 90 min (Cedarbaum, 1987). This strategy increases the bioavailability of LD in the central nervous system but also allows for lower dosing, thereby reducing peripheral side effects such as nausea and hypotension (Rinne and Molsa, 1979; Hauser, 2009; Salat and Tolosa, 2013). Since its introduction in the 1970s, LD + DCIs have remained the gold standard in PD treatment due to its remarkable efficacy in alleviating motor symptoms (LeWitt and Fahn, 2016).

Patients often experience significant improvements in their ability to perform daily activities, as LD + DCIs effectively mitigates the debilitating symptoms of PD. However, prolonged use of LD therapy leads to non-physiological stimulation of dopamine receptors, which is consequently linked to motor complications such as motor fluctuations and dyskinesia (Obeso et al., 2000; Dewey, 2004). Motor complications frequently occur in early PD, with a 30%–40% incidence within 5 years of treatment, and the number accumulates to 60% by the 10th year as the disease progresses (Ahlskog and Muenter, 2001; Turcano et al., 2018; Prange et al., 2019). Some patients develop motor fluctuations, such as the “wearing-off” phenomenon, where the drug’s effects become transient and less durable. Moreover, patients may experience sudden “off” periods, where they switch unpredictably from an ‘on’ state of mobility to a significantly impaired ‘off’ state, known as the “on-off phenomenon.” Additionally, during the “freezing phenomenon,” patients may suddenly feel as though their feet are glued to the ground, making it difficult to initiate movement, especially in situations like turning or passing through narrow spaces. What’s more, involuntary movements known as dyskinesias can emerge, further complicating the clinical management of PD and diminishing patients’ quality of life (Chapuis et al., 2005).

The development of motor complications associated with oral LD therapy is a complex process influenced by multiple factors. These include the pulsatile stimulation of dopamine receptors due to fluctuating LD levels, the progressive degeneration of dopaminergic neurons, and individual patient characteristics such as genetic predispositions and disease severity (Bhidayasiri and Truong, 2008; Warren Olanow et al., 2013; Ray Chaudhuri et al., 2018). While BSZ and CD both aim to enhance LD efficacy by inhibiting its peripheral conversion, they contributed to different pharmacokinetic profiles of LD (Iwaki et al., 2015). These differences may affect the incidence and severity of motor complications. Therefore, we hypothesized that BSZ and CD may impact the emergence of these complications in a differential manner.

In this pharmacovigilance study, we seek to comprehensively analyze the safety profiles of BSZ and CD in combination with LD by utilizing data from the FDA Adverse Event Reporting System (FAERS) database. This database provides a valuable repository of spontaneous adverse event reports, offering insights into the real-world experiences of patients and healthcare providers (Poluzzi et al., 2012). Our objective was to systematically review the risk of levodopa-induced motor complications associated with BSZ and CD in PD and provide a reference for clinical decision making.

## 2 Methods

### 2.1 Study design and data source

The present study was a retrospective pharmacovigilance analysis aimed at comparing the adverse events (AEs) profiles associated with levodopa-induced motor complications in the context of BSZ and CD use in PD patients. Data were extracted from the FAERS database, which is a publicly accessible repository designed to support post-marketing safety surveillance of drug and therapeutic biologic products. The FAERS database contains spontaneous reports from consumers, healthcare professionals, drug manufacturers, and other non-healthcare workers. The data used in this analysis spanned from the first quarter of 2004 to the second quarter of 2024.

### 2.2 Data extraction and inclusion criteria

AEs refer to any untoward medical occurrences in a patient or clinical trial participant. In contrast, Adverse event reports (AERs) are documented reports of AEs submitted by healthcare professionals, patients, or other stakeholders to pharmacovigilance systems. While AEs represent the actual clinical events, AERs are the formalized documentation and reporting of these events. We included AERs that mentioned levodopa in combination with either benserazide or carbidopa. The reports were filtered based on the preferred terms (PTs) from the Medical Dictionary for Regulatory Activities (MedDRA) to ensure standardization of the AE terminology. We extracted demographic information such as patient age, gender, and the date the reports were received, as well as details regarding the seriousness of the AEs, including hospitalization, disability, life-threatening events, and death.

### 2.3 Adverse event and drug identification

To identify records of the target drugs, we performed text string searches using both brand and generic names. We extracted AEs marked with ‘levodopa’ in combination with ‘benserazide’ or ‘carbidopa’ as the suspected drugs. We reviewed individual safety reports and counted records according to PTs. Two independent researchers, one with expertise in pharmacovigilance and the other in movement disorders, classified the AERs and collected clinical characteristics of the patients, including gender, age, and AE outcomes. To reduce bias caused by drug delivery, only AERs due to oral administration of drugs were included. To minimize indication bias, we analyzed cases with PD as the primary indication and excluded those listing PD as an adverse event. Given a considerable proportion of reporters was PD patients, cases with dementia and psychosis due to PD were excluded. With focus on the motor complications, we selected two system organ classes (SOC) associated with motor fluctuations as well as dyskinesia for further analysis, which are presented as follows: nervous system disorders/general disorders and administration site conditions. Apart from two complications with corresponding PTs in the database, we reclassified the following terms: dyskinesia and wearing-off. Dyskinesia (Gupta et al., 2024) was searched and defined using key words: dyskinesia, dystonia, hyperkinesia, ballismus, and alien limb syndrome. Wearing-off was defined using incomplete or shortened therapeutic response. A total of 4 specific motor complications was of interest, including on and off phenomenon, freezing phenomenon, dyskinesia, and wearing-off.

### 2.4 Statistical analysis

We employed disproportionality analysis to detect and assess AE signals associated with carbidopa and benserazide. The reporting odds ratio (ROR) was calculated to measure the association ratio of observed frequency in the exposed population to the non-exposed (Ma et al., 2023). These metrics indicate the strength of the AE signal, with higher values suggesting a stronger statistical relationship between the drug and the AE. To confirm the AE signals and reduce false positives, we also applied the Bayesian confidence propagation neural network (BCPNN) and the empirical Bayesian geometric mean (EBGM) (Ma et al., 2023; Bate et al., 1998; Du et al., 2024). A signal was deemed significant if it met the following criteria: ROR ≥3 with a 95% CI lower bound >1, IC 95% CI lower bound >0, and EBGM 95% CI lower bound >2. Supplementary Table S1 illustrates the concepts of disproportionate measurement and the criteria for signal detection.

All analyses were conducted using R 4.3.2 software and data visualization was performed. Categorical data were expressed as counts and percentages, continuous data were presented using median and IQR. Furthermore, the RORS of AEs on BSZ and CD were visualized using forestplots. While a greater ROR suggests higher risk of AE, absence of overlaps in CI of the RORs in both drugs indicates statistical significance for comparison. Further inter-group comparisons were conducted using chi-square and fisher tests, and two-sided p values less than 0.05 were consider statistical significance.

## 3 Results

### 3.1 Data source and screening

**FIGURE 1:**
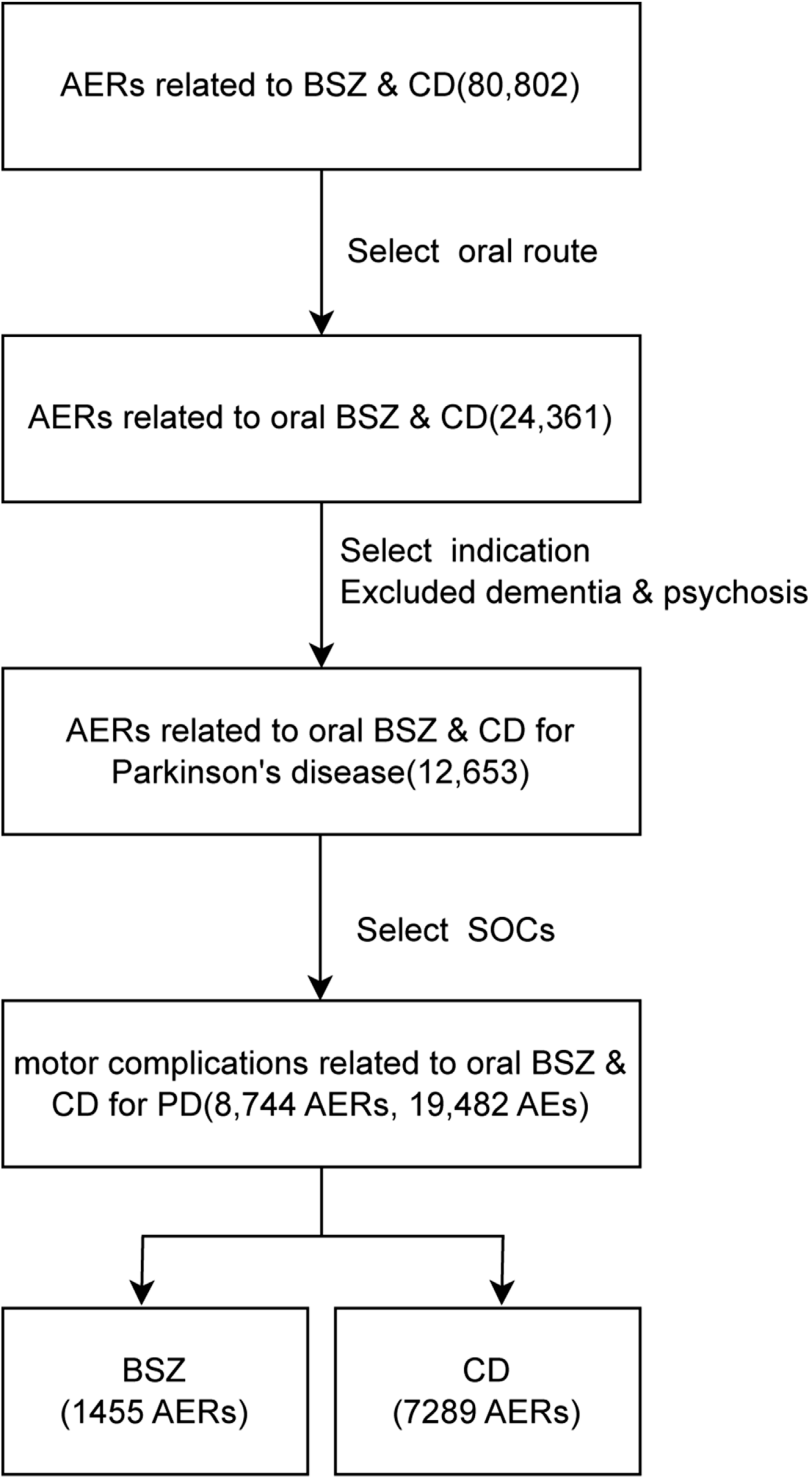
Flowchart of data screening.

From the first quarter of 2004 to the second quarter of 2024, a total of 80,802 AERs was identified in the use of BSZ and CD in combination of levodopa, out of which 24,361 AERs were documented in the oral administration of these drugs. These AERs were further narrowed to 12,653 reports involving the treatment of PD, excluding those with comorbid dementia or psychosis. For analysis of systems related to motor complications, we selected two soc: ‘nervous system disorder’, as well as ‘general disorders and administration site conditions’. Our screening in the FAERS database yielded a total of 1,455 AERs associated with BSZ and 7,289 AERs associated with CD in the context of oral levodopa use for PD. The flowchart of the data screening process was shown in Figure 1.

### 3.2 Descriptive analysis

Results of descriptive Analysis were presented in Table 1. The demographic characteristics of the reports revealed that the majority of patients were aged 50 years or older, with a significant number of reports coming from individuals aged 65 and above for both BSZ (63.92%) and CD (51.97%). For juvenile Parkinson’s (age<20) (Niemann and Jankovic, 2019), there were 2 reports associated with BSZ and CD, respectively. For early-onset Parkinson’s (20–50 years old) (Gibb and Lees, 1988), there were 26 reports associated with benserazide, constituting 1.79% of all BSZ-related reports. For carbidopa, the number was higher with 147 reports, which is 2.02% of all CD-related reports. The average weight of patients reported was 67.00 kg (IQR: 55.25, 77.00) for BSZ and 70.30 kg (IQR: 58.96, 83.90) for CD.

**TABLE 1:** Characteristics of AERs related to motor complications on oral BSZ and CD in PD.

Variable	BSZ (N = 1,455)	CD (N = 7,289)
Age		
<20	2 (0.14)	2 (0.03)
20–50	26 (1.79)	147 (2.02)
50–65	178 (12.23)	945 (12.96)
≥65	930 (63.92)	3,788 (51.97)
unknow	319 (21.92)	2,407 (33.02)
Reporter		
Physician	407 (27.97)	999 (13.71)
Consumer	388 (26.67)	5,127 (70.34)
Other health-professional	303 (20.82)	528 (7.24)
Pharmacist	298 (20.48)	519 (7.12)
unknown	59 (4.05)	113 (1.55)
Lawyer		3 (0.04)
Serious Outcomes		
hospitalization	906 (46.25)	1790 (39.16)
other serious	649 (33.13)	1843 (40.32)
life threatening/death	353 (18.02)	709 (15.51)
disability	50 (2.55)	228 (4.99)
congenital anomaly	1 (0.05)	1 (0.02)
Reported countries/districts		
Europe	675 (46.39)	650 (8.92)
United States		5,141 (70.53)
Japan	78 (5.36)	248 (3.40)
South America	65 (4.47)	50 (0.69)
other	637 (43.78)	1,200 (16.46)
Sex		
female	633 (43.51)	3,429 (47.04)
male	789 (54.23)	3,703 (50.80)
unknown	33 (2.27)	157 (2.15)
Weight	67.00 (55.25,77.00)	70.30 (58.96,83.90)

BSZ, benserazide; CD, carbidopa.

**FIGURE 2:**
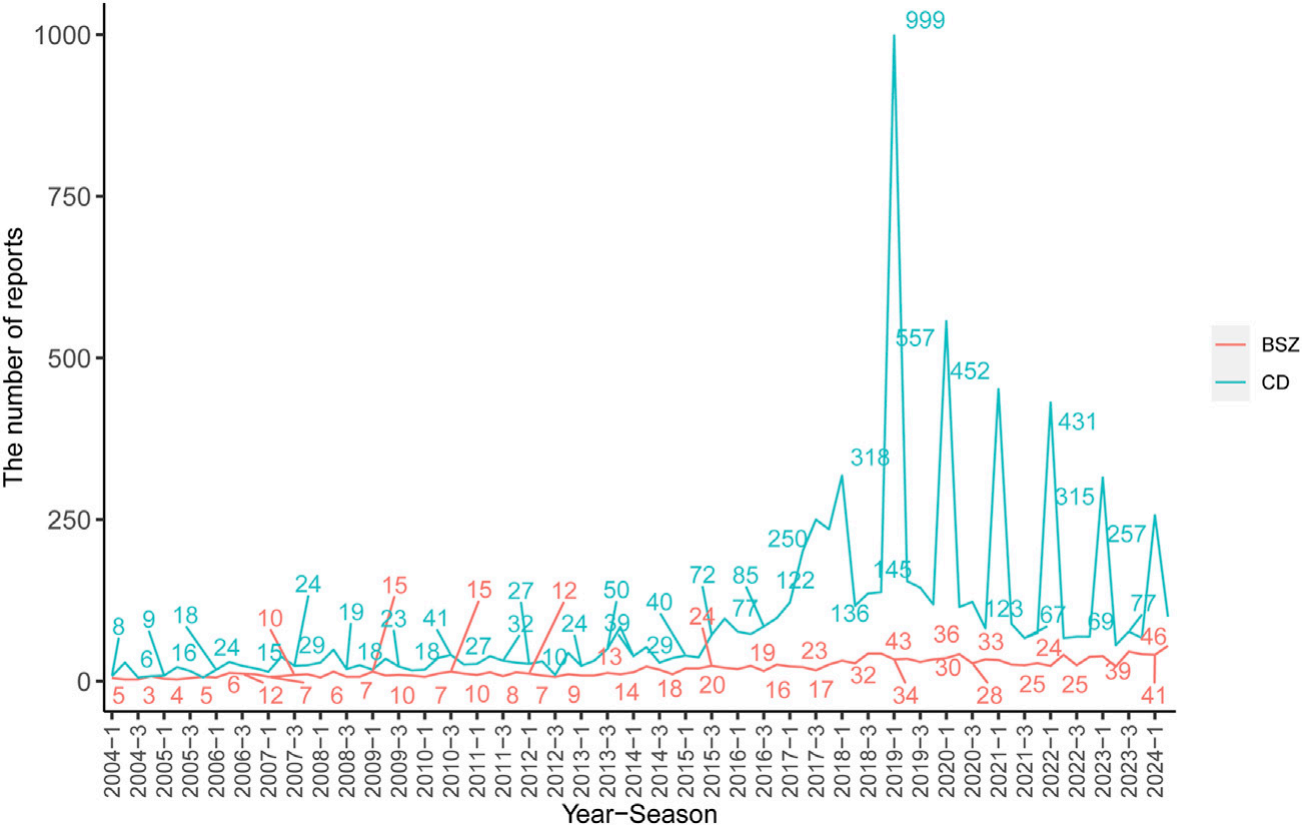
Number of AERs in each year and season for oral BSZ and CD in PD.

In terms of reporter types, consumers were the most frequent reporters for both BSZ (26.67%) and CD (70.34%), followed by physicians and other health professionals. The serious outcomes of the AEs were presented, with hospitalization being the most common outcome for both BSZ (46.25%) and CD (39.16%), followed by other serious outcomes, life-threatening conditions/death, and disability. Geographically, the majority of CD reports originated from the United States (70.53%), followed by European countries (8.92%) and South American countries (0.69%). In contrast, BSZ reports were predominantly from European countries (46.39%) and Japan (5.36%). The number of AERs in each year and quarter for BSZ and CD was presented in Figure 2.

### 3.3 Analysis of concomitant anti-parkinsonian drugs in the occurrence of motor complications

Table 2 presents the top 5 concomitant anti-Parkinsonian drugs reported in association with adverse events for benserazide (BSZ) and carbidopa (CD). The analysis reveals the co-occurrence of other medications used in the treatment of PD and their potential to contribute to or be associated with the reported adverse events.

**TABLE 2:** Top 5 concomitant anti-parkinsonian drugs in adverse events reports.

BSZ (N, %)	CD (N, %)
pramipexole (254, 2.61)	pramipexole (811, 2.50)
amantadine (107, 1.10)	amantadine (522, 1.61)
rasagiline (102, 1.05)	entacapone (500, 1.54)
ropinirole (97, 1.00)	rasagiline (498, 1.54)
rotigotine (94, 0.97)	ropinirole (383, 1.18)

BSZ, benserazide; CD, carbidopa.

For benserazide, the top concomitant anti-Parkinsonian drug reported was pramipexole (Antonini et al., 2010), with 254 reports representing 2.61% of all BSZ-related adverse events. This was followed by amantadine (Schwab et al., 1969) with 107 reports (1.10%), rasagiline (Oldfield et al., 2007) with 102 reports (1.05%), ropinirole (Lieberman et al., 1998) with 97 reports (1.00%), and rotigotine (Reynolds et al., 2005) with 94 reports (0.97%). In the case of carbidopa, amantadine was the second most reported with 522 reports (1.61%), followed by entacapone (Schrag, 2005) with 500 reports (1.54%), rasagiline with 498 reports (1.54%), and ropinirole with 383 reports (1.18%).

### 3.4 Adverse event profiles

Table 3 provides a detailed analysis of the motor complications associated with the use of BSZ and CD in the context of levodopa treatment for PD. The most frequently reported motor complication was dyskinesia, with a total of 1,566 reports, accounting for 7.99% of all motor complication reports. Specifically, there were 324 reports associated with BSZ (9.16%) and 1,214 reports with CD (7.71%). This suggests that dyskinesia is a common issue for both groups, but it is slightly more prevalent in the BSZ group. The on and off phenomenon, characterized by fluctuations in motor performance, was reported in 418 cases (2.15%) of the total. BSZ was associated with 157 reports (4.2%), while CD had 261 reports (1.66%). Freezing phenomenon, or the sudden inability to move, was reported in 356 cases (1.83%) overall. There were 77 reports associated with BSZ (2.06%) and 279 reports with CD (1.77%). Wearing-off, which refers to the diminishing effect of medication over time, was reported in 724 cases (3.72%). BSZ had 64 reports (1.71%), while CD had a higher number with 660 reports (4.19%). This indicates that the perception of wearing-off is more common in the CD group. Further, subgroup analysis suggests that dyskinesia is more prevalent in female patients than male in both BSZ (P < 0.05) and CD (P < 0.001), with an approximate increase of 2% in report ratios.

**TABLE 3:** Motor complications associated with the use of BSZ and CD.

Motor complication		Dyskinesia (N,%)	Wearing_off (N,%)	On and off phenomenon (N,%)	Freezing phenomenon (N,%)
Total reports		1,556 (7.99)	724 (3.72)	418 (2.15)	356 (1.83)
BSZ	sutotal_BSZ	342 (9.16)	64 (1.71)	157 (4.2)	77 (2.06)
	female	163 (10.65)*	25 (1.63)	51 (3.33)	27 (1.76)
	male	175 (8.16)	39 (1.82)	105 (4.9)*	49 (2.29)
	unknown	4 (6.67)		1 (1.67)	1 (1.67)
CD	sutotal_CD	1,214 (7.71)	660 (4.19)	261 (1.66)	279 (1.77)
	female	666 (8.67)***	347 (4.52)	113 (1.47)	112 (1.46)
	male	527 (6.79)	308 (3.97)	148 (1.91)*	166 (2.14)*
	unknown	21 (6.89)	5 (1.64)		1 (0.33)

BSZ, benserazide; CD, carbidopa.

*p < 0.05.

***p < 0.001.

### 3.5 Disproportionality analysis

Table 4 presents the results of the disproportionality analysis for motor complications associated with BSZ and CD use in the context of levodopa treatment for PD. The table highlights the safety signals for specific motor complications, including dyskinesia, on and off phenomenon, freezing phenomenon, wearing-off, as identified by various statistical measures.

**TABLE 4:** Disproportionality analysis for motor complications associated with BSZ and CD use in the context of levodopa treatment for PD.

Motor complication	BSZ				CD			
	N	ROR (95% CI)	IC(IC025)	EBGM(EBGM05)	N	ROR (95% CI)	IC(IC025)	EBGM(EBGM05)
Nervous system disorders								
Dyskinesia	342	16.5 (14.76, 18.45)	3.91 (3.75)	15.03 (13.69)	1,214	13.81 (13.02, 14.65)	3.66 (3.58)	12.65 (12.04)
On and off phenomenon	157	170.74 (145.03, 201.01)	7.29 (7.06)	156.65 (136.65)	261	67.5 (59.46, 76.63)	5.95 (5.77)	61.75 (55.53)
Freezing phenomenon	77	104.35 (83.01, 131.17)	6.64 (6.31)	99.47 (82.14)	279	96.36 (85.07, 109.14)	6.42 (6.24)	85.46 (77)
General disorders and administration site conditions								
Wearing_off	64	3.03 (2.37, 3.88)	1.58 (1.23)	3 (2.44)	660	7.66 (7.08, 8.28)	2.87 (2.76)	7.33 (6.86)

BSZ, benserazide; CD, carbidopa.

Dyskinesia was identified as a significant safety signal for both BSZ and CD. BSZ had 342 case reports with a Reporting Odds Ratio (ROR) of 16.5 (95% CI 14.76, 18.45), and an Information Component (IC) of 3.91(IC025 3.75). The corresponding Expected Bayesian Geometric Mean (EBGM) was 15.03 (EBGM05 13.69). For CD, there were 1,214 case reports, with an ROR of 13.81 (95% CI 13.02, 14.65), an IC of 3.66(IC025 3.58), and an EBGM of 12.65 (EBGM05 12.04). These high values indicate a strong statistical association between both drugs and dyskinesia, with a higher risk in patients treated with BSZ.

The on and off phenomenon also showed strong safety signals for BSZ, with 157 case reports, an ROR of 170.74 (95% CI 145.03, 201.01), an IC of 7.29 (IC025 7.06), and an EBGM of 156.65 (EBGM05 136.65). For CD, there were 261 case reports, with an ROR of 67.5 (95% CI 59.46, 76.63), an IC of 5.95 (IC025 5.77), and an EBGM of 61.75 (EBGM05 55.53). Likewise, the higher ROR and IC values for BSZ suggest a stronger association with the on and off phenomenon compared to CD.

Freezing phenomenon was another significant safety signal, with BSZ having 77 case reports, an ROR of 104.35 (95% CI 83.01, 131.17), an IC of 6.64 (IC025 6.31), and an EBGM of 99.47 (EBGM05 82.14). CD had 279 case reports, with an ROR of 96.36 (95% CI 85.07, 109.14), an IC of 6.42 (IC025 6.24), and an EBGM of 85.46 (EBGM05 77). Both drugs showed a strong association with the freezing phenomenon, as indicated by the high ROR and IC values.

Lastly, wearing-off was identified as a safety signal, particularly for CD, with 660 case reports, an ROR of 7.66 (95% CI 7.08, 8.28), an IC of 2.87 (IC025 2.76), and an EBGM of 7.33 (EBGM05 6.86). BSZ had 64 case reports, with an ROR of 3.03 (95% CI 2.37, 3.88), an IC of 1.58 (IC025 1.23), and an EBGM of 3 (EBGM05 2.44). The higher ROR and IC values for CD suggest a stronger association with wearing-off compared to BSZ.

### 3.6 Comparison of safety signals

**FIGURE 3:**
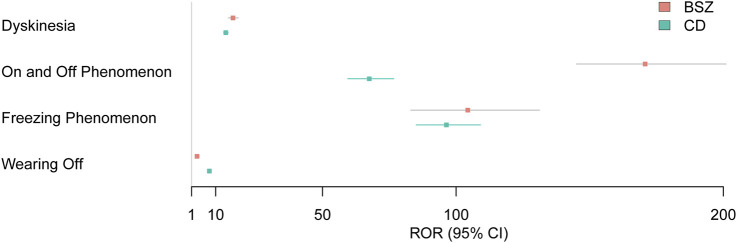
Forest plot presenting RORs with 95% CIs for motor complication signals associated with BSZ and CD.

Figure 3 presents a forest plot visualizing the Reporting Odds Ratios (RORs) with 95% confidence intervals (CIs) for selected motor complications associated with BSZ and CD. This graphical representation provides a clear overview of the strength of the association between each motor complication and the two drugs, allowing for direct comparison and assessment of their relative risks. Dyskinesia shows a high ROR for both BSZ and CD, with BSZ having an ROR of 16.5 (95% CI 14.76, 18.45) and CD with an ROR of 13.81 (95% CI 13.02, 14.65) (P < 0.001). These elevated RORs indicate a strong association between both drugs and the risk of dyskinesia, with BSZ showing a sightly higher risk for dyskinesia. The on and off phenomenon demonstrates an even more pronounced ROR for BSZ at 170.74 (95% CI 145.03, 201.01) compared to CD with an ROR of 67.5 (95% CI 59.46, 76.63) (P < 0.001). This suggests that the risk of experiencing the on and off phenomenon is considerably higher for BSZ users. Freezing phenomenon also exhibits high RORs for both drugs, with BSZ at 104.35 (95% CI 83.01, 131.17) and CD at 96.36 (95% CI 85.07, 109.14). These values highlight the strong association between both drugs and the risk of freezing phenomenon. Lastly, wearing-off is particularly notable for CD, with an ROR of 7.66 (95% CI 7.08, 8.28), indicating a significantly higher risk of wearing-off among CD users compared to BSZ, which has an ROR of 3.03 (95% CI 2.37, 3.88) (P < 0.001).

## 4 Discussion

The present pharmacovigilance study of the FAERS database aimed to compare the safety profiles of BSZ and CD when used in conjunction with LD for the treatment of PD. We identified a total of 8,744 AERs related to motor complications, where 19,482 AEs were recorded. The present study highlighted the AEs related to motor complications as follows: dyskinesia, on-off phenomenon, freezing phenomenon, and wearing-off, all of which was identified to be associated with oral administration of both BSZ and CD by disproportionality analysis using three different methods. Further analysis of the statistical measures, particularly RORs, revealed distinct safety signals associated with each DCI, with BSZ showing a higher risk of certain motor complications compared to CD. Specifically, we identified stronger signals for dyskinesia, and on-off phenomenon with BSZ, while CD was more strongly associated with wearing-off.

Our subgroup analysis found that dyskinesia was significantly more prevalent in female patients than in male patients across both drug groups. This observation aligns with studies on levodopa pharmacokinetics, which indicate that women tend to have higher plasma concentrations of levodopa, as reflected by significantly higher area under curve (AUC) and maximum plasma concentration (Cmax) compared to men (Conti et al., 2022; Contin et al., 2022). For instance, Conti et al. (2022) found that female sex significantly predicted AUC and Cmax by assessing levodopa pharmacokinetics in 35 patients with PD at their first-ever intake of levodopa (Conti et al., 2022). Similarly, Conti et al. (2022) demonstrated that the AUC for levodopa was 27% higher in females than in males using data from a large series of levodopa therapeutic monitoring (Contin et al., 2022). Furthermore, the same study indicated that female patients required a 25% lower weight-normalized dose of levodopa to achieve the same bioavailability as their male counterparts (Contin et al., 2022). These pharmacokinetic differences likely contribute to the increased risk of dyskinesia observed in female patients. The findings underscore the importance of considering sex when assessing the risk of dyskinesia in PD patients treated with levodopa.

The pharmacokinetic differences of LD observed when combined with BSZ or CD provide valuable mechanistic insights into the varying safety profiles of these DCIs. As detailed in the study by Da Prada et al. (1987), the combination of levodopa with BSZ results in more rapid elevation and reduction of LD concentration compared to CD. Additionally, the study by Shiraishi et al. (2020) highlights the significance of high levodopa plasma concentrations in the development of levodopa-induced dyskinesia (LID) in patients with PD. The findings suggest that a higher Cmax and AUC are associated with an increased risk of LID, which aligns with the pharmacokinetic characteristics of BSZ (Iwaki et al., 2015), potentially explaining the higher incidence of dyskinesia observed with this combination. The rapid increase, high Cmax, and subsequent drop in levodopa levels, as seen with BSZ, result in a more pulsatile and excessive dopaminergic stimulation, potentially leading to a greater risk of LID and on-off phenomenon. On the other hand, the relatively limited potency of CD (Iwaki et al., 2015) might contribute to higher risks of wearing-off.

The comparison of two DCIs, BSZ and CD, in combination with levodopa has a history dating back to the 1970s, with the majority of studies focusing on their efficacy and short-term complications (Greenacre et al., 1976; Rinne and Mölsä, 1979). While these investigations have not revealed statistically significant differences in effectiveness, there has been a noted patient preference for one over the other due to variations in individual responses. Some researcher suggested combined use of BSZ and CD may be useful for individuals with PD (Lieberman et al., 1984). When it comes to long-term complications, such as motor fluctuations, there is a dearth of research. A notable exception is a retrospective cohort study from Japan (Baba et al., 2022) that analyzed 52 patients and concluded that LD/CD therapy with a CD to LD ratio of 1:10 might delay the onset of motor fluctuations compared to LD/BSZ therapy with a ratio of 1:4. This finding highlights the potential impact of LD/DCI dosing ratio on disease progression in PD. However, the effects of different selection of DCIs could not be established. With the focus on DCI components, our study revealed distinct motor complication signals in a real-world setting by leveraging the FAERS database. The large sample size and the use of disproportionality analysis algorithms allow for detection and assessment of specific motor complications between BSZ and CD in combination with oral LD, offering new insight for the clinical management of PD. Consistent with previous studies (Iwaki et al., 2015; Baba et al., 2022; Chaudhuri et al., 2013), the present study indicates LD/BSZ can be beneficial for individuals needing rapid relief from motor symptoms, and LD/CD might be advantageous for those experiencing significant dyskinesia due to overstimulation of dopamine within the striatum. Further, the results indicate those with unpredictable off episodes could consider switching their oral levodopa formula from LD/BSZ to LD/CD.

The core concept in the management of PD is the principle of continuous dopaminergic stimulation, which is designed to maintain a stable and therapeutic level of dopamine in the brain to prevent and manage motor complications. Despite various pharmaceutical advancements and innovative delivery methods for levodopa, including intrajejunal (Olanow et al., 2014) and subcutaneous infusion (Soileau et al., 2022), oral administration of LD/DCI remains the mainstay in clinical practice. However, clear guidelines on the selection of oral LD/DCIs have not been established. Our study, therefore, holds significant value as it offers a comparative analysis of motor complication profiles of BSZ and CD, the two most frequently used DCIs in conjunction with oral LD. To our knowledge, the present study is the first pharmacovigilance analysis of the FAERS database concerning levodopa-induced motor complications associated with BSZ and CD in PD. With the drugs orally administered, the confounding effects of levodopa delivery could be excluded. However, different formation of LD + DCIs could also influence AERs in both BSZ and CD due to distinct LD pharmacokinetics, which awaits exploration in the future.

The present study is not without limitations. Firstly, the FAERS database, while comprehensive, is subject to reporting biases, and the lack of standardized grading of AEs may limit the accuracy of our risk estimates. Secondly, the spontaneous nature of the reports means that our findings cannot be used to establish causality but rather to generate hypotheses that require further investigation in controlled studies. Thirdly, the interactions between the selected medication and concomitant drugs awaits further investigation, as patients with PD often take multiple anti-parkinsonian drugs simultaneously. Lastly, the cumulative levodopa equivalent daily dose (LEDD) (Jost et al., 2023) could not be calculated in each case report, so that the dose-effect relations was not evaluated. Therefore, our results should be interpreted with cautions. Future research should focus on prospective, randomized studies comparing BSZ and CD in terms of their impact on motor complications in PD. Further exploration of the pharmacokinetic and pharmacodynamic differences between these two DCIs, as well as their impact on the progression of PD, is warranted. Such studies will provide a more definitive understanding of the comparative safety and efficacy of BSZ and CD, guiding clinicians in the selection of the most appropriate DCI for individual patients with PD.

## 5 Conclusion

In conclusion, our disproportionality analysis of the FAERS database highlights the differential motor complication profiles of BSZ and CD when co-administered with levodopa in the treatment of PD. The findings suggest that BSZ is more strongly associated with dyskinesia and on-off phenomenon, while CD is linked to a higher risk of “wearing-off”. These results underscore the importance of choosing DCIs when tailoring treatment plans for patients with PD, as this choice may influence the incidence of motor complications. Further research is needed to elucidate the mechanisms underlying these differences and to optimize the management of motor complications in PD.

## Data Availability

Publicly available datasets were analyzed in this study. This data can be found here: FAERS database.
